# Additive Effects of Lithium Salts with Various Anionic Species in Poly (Methyl Methacrylate)

**DOI:** 10.3390/molecules26134096

**Published:** 2021-07-05

**Authors:** Asae Ito, Koh-hei Nitta

**Affiliations:** Polymer Physics Laboratory, Institute of Science and Engineering, Kakuma Campus, Kanazawa University, Kanazawa 920-1192, Ishikawa, Japan; asae@se.kanazawa-u.ac.jp

**Keywords:** poly (methyl methacrylate), lithium salts, dynamic mechanical properties

## Abstract

We report that lithium salts in lithium-ion batteries effectively modify the physical properties of poly (methyl methacrylate) (PMMA). The glass transition temperature (*T_g_*) is an indicator of the heat resistance of amorphous polymers. The anionic species of the salts strongly affected the glass transition behavior of PMMA. We focused on the additive effects of various lithium salts, such as LiCF_3_SO_3_, LiCOOCF_3_, LiClO_4_, and LiBr, on the *T_g_* of PMMA. The large anions of the former three salts caused them to form macroscopic aggregates that acted as fillers in the PMMA matrix and to combine the PMMA domains, increasing *T_g_*. On the other hand, LiBr salts dispersed microscopically in the PMMA matrix at the molecular scale, leading to the linking of the PMMA chains. Thus, the addition of LiBr to PMMA increased *T_g_* as well as the relaxation time in the range of glass to rubber transition.

## 1. Introduction

Transparent plastics, in particular lightweight and highly processable poly(methyl methacrylate) (PMMA), have replaced various inorganic glassy materials in many optical components, such as optical films for displays, optical lenses, optical fibers, and touch panels. PMMA, in particular, has the best transparency among the existing transparent plastics. The ester groups in PMMA make it possible to modify the physical properties of acrylic resins by copolymerization with other monomers or by modifying them with other chemical species [1,2,3,4,5].

Ionomers, which are ionic polymers with a small amount of ionic groups introduced into polymer chains, are also effective in the modification of PMMA using polar groups. Interestingly, these ionic groups aggregate in the host polymer matrix to form microphase-separated structures, which directly affect their molecular mobility. The introduction of a small amount of ionic groups into polymers, such as ionomers, has been attempted since the 1950s [6]; accordingly, a detailed characterization of their molecular aggregation states has been strongly desired. The formation of such ionic aggregates is considered to have a direct effect on the physical and mechanical properties of ionomers. However, since ionomers are complicated multi-body systems [7], it is difficult to elucidate the relationship between the molecular interactions in them and their mechanical properties.

We recently developed a simple method for modifying PMMA by adding salts. This method was inspired by the solid electrolyte system of lithium-ion batteries. Poly(ethylene oxide) (PEO), which is a rubbery polymer at room temperature, has been conventionally used as a solid electrolyte material [8,9]. It is well known that the addition of lithium ions increases the glass transition temperature (*T_g_*) of PEO, and this phenomenon has been recognized as a problem for batteries because the increase in *T_g_* inhibits the conduction of ions. This seems to be owing to the ion-dipole interaction between the oxygen atoms in PEO and the lithium cations, leading to the suppression of the segmental motion of PEO chains [10,11]. Furthermore, the electrolytes based on PMMA have also been reported [12,13,14,15].

In light of the above, we investigated the additive effects of lithium salts on the glass transition behavior of PMMA, based on the intermolecular interaction between the ester groups of the PMMA side chains [16] and the added lithium salts [17,18,19]. To date, we have succeeded in reducing the birefringence [17] and improving the heat resistance [18] of transparent plastics by adding lithium salts to PMMA and utilizing the intermolecular interaction between the polar groups of PMMA and the added salts. The objective of this work was to clarify the relationship between the salt aggregation structure and the molecular mobility of PMMA chains in PMMA/salt-doped systems. For this purpose, we investigated the additive effects of lithium salts with various anions on the dynamic mechanical spectra of the hosting PMMA matrix.

## 2. Results and Discussion

### 2.1. Effects of the Anion Characteristics of Salts

**Figure 1:**
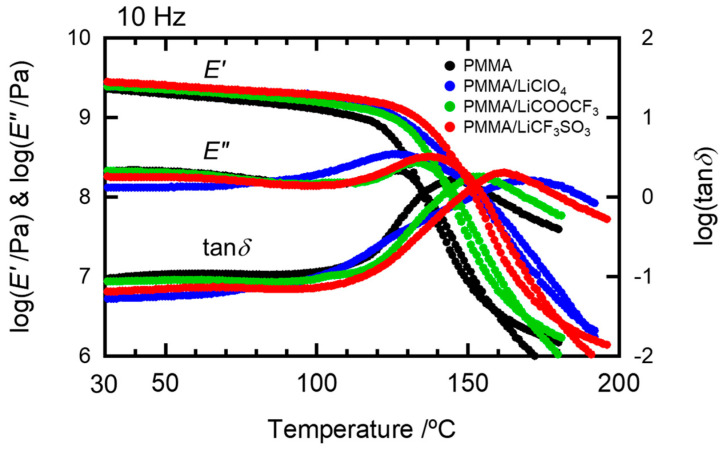
The dynamic mechanical properties of the prepared PMMA/salt samples with [Li]/[C=O] = 0.07.

We examined the dynamic mechanical spectra of the PMMA sheets doped with several lithium salts with large anions: LiCF_3_SO_3_, LiCOOCF_3_, and LiClO_4_. These lithium salts have been used as typical fillers for lithium electrolytes. The resulting dynamic mechanical properties of these samples for temperatures in the 30–200 °C range and salt content of 7 mol% are shown in Figure 1. In addition, the *T_g_* values of the PMMA matrix, estimated from the peak temperature of the *E*” curve, are summarized in Table 1. The *T_g_* values measured at 10 Hz were different from previous differential scanning calorimetry (DSC) data [18,19] but showed similar behavior as DSC. This difference in values is because the DSC data results from the heat capacity changes under the glass transition, whereas the dynamic mechanical data are associated with the mechanical relaxation due to molecular motion and depend on the mechanical frequency. These data indicate that doping with lithium salts increases the *T_g_* values of the PMMA matrix. It is likely that the obvious increase in *T_g_* is caused by the molecular interaction between the lithium cations and ester groups in PMMA. This is because the molecular interactions between the cations and ester groups in the present PMMA/salt samples were confirmed by the ATR spectra for wavenumbers in the 1650–1780 cm^−1^ range, as shown in Figure S2.

**Figure 2:**
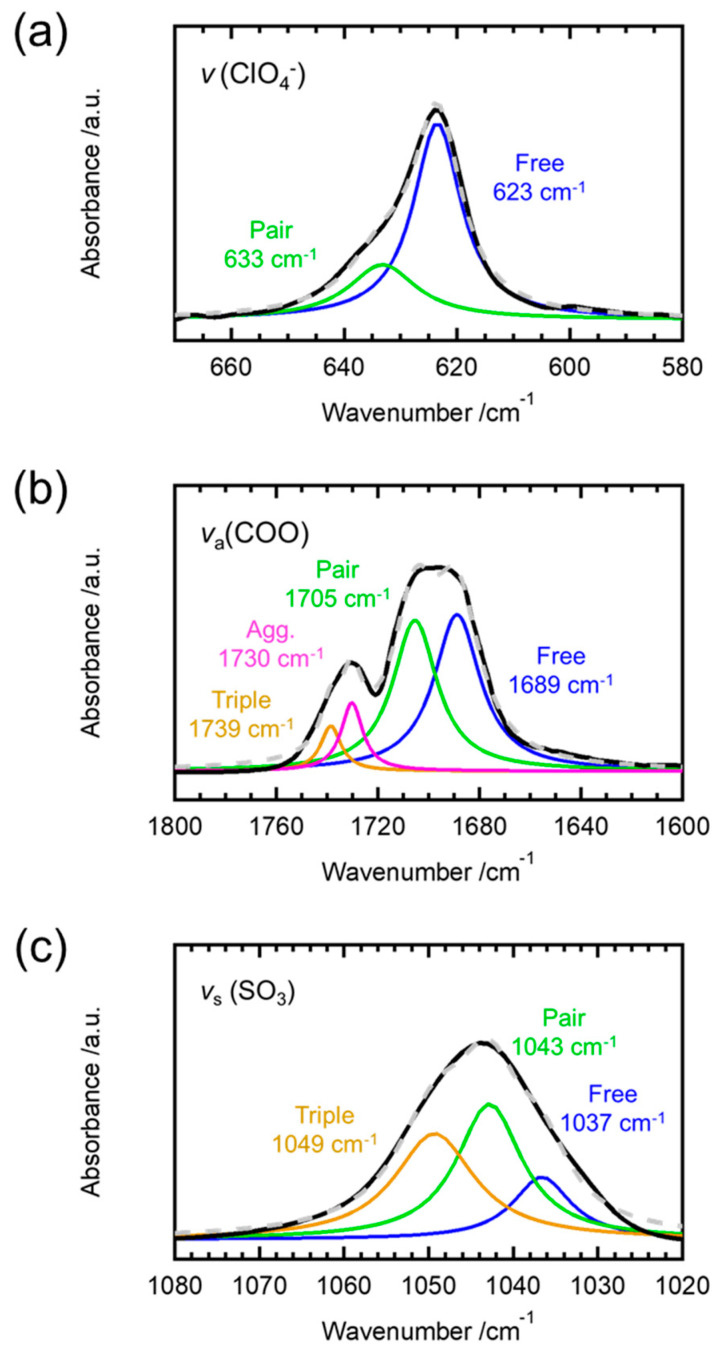
ATR spectra of the PMMA samples doped with (**a**) LiClO_4_, (**b**) LiCOOCF_3_, and (**c**) LiCF_3_SO_3_. Their concentrations of lithium salts are [Li]/[C=O] = 0.07. The black lines denote the experimental spectra and the dashed gray lines denote the computational ones.

We decomposed the corresponding peaks of the ATR spectra and estimated the concentration of PMMA/salt aggregates based on their peak areas to better determine the effects of the anionic species of the added salts on the *T_g_* of PMMA. The deconvoluted ATR spectra of the different PMMA/salt samples are shown in Figure 2, fitted by Lorentzian functions [20].

In Figure 2a, the stretching vibration modes of ClO_4_ are separated, yielding the peaks at 623 cm^−1^ and 635 cm^−1^, corresponding to the free anions and bound or contact ClO_4_^−^ anions [21,22], respectively. Figure 2b shows the peak deconvolution of the ATR spectra of the PMMA/LiCOOCF_3_ sample with the added salt at 7 mol%. Here, we removed the pristine PMMA spectrum from the ATR spectrum of its doped spectrum because the bands ascribed to the anions in the 1700 cm^−1^ band overlapped with the carbonyl symmetric stretching vibration mode of pristine PMMA at 1724 cm^−1^. According to Regis et al. [23], the asymmetric stretching vibration band of COO^−^ is composed of four modes, at 1695 cm^−1^ (free ions), 1736 cm^−1^ (triplet anions), 1710 cm^−1^ (ion pairs), and 1729 cm^−1^ (aggregates). Figure 2c shows the peak decomposed ATR spectra of PMMA/LiCF_3_SO_3_ with 7 mol% of the added salt. In addition, we removed the PMMA spectra from the ATR spectra of the doped samples because the PMMA peaks for wavenumbers in the 1020–1080 cm^−1^ range overlapped. The symmetric stretching vibration mode of SO_3_^−^ is composed of three modes at 1032 cm^−1^ (free ions), 1040 cm^−1^ (ion pairs), and 1045 cm^−1^ (triplet anions) [24]. The contributions of the different anionic species (ClO_4_^−^, CF_3_SO_3_^−^, and COOCF_3_^−^) were quantitatively estimated from the peak areas of the decomposed spectra (Table 2).

Here, we considered the fraction of non-free anions as the aggregation degree. Solubility parameters were evaluated by estimating the cohesive energies of these salts using the atomic cluster contribution method. As shown in the Supporting Information, the fraction of non-free anions is related to the solubility parameter, strongly suggesting that the fraction of non-free anions can be considered the level of the aggregation state of anions in the doped PMMA samples. The aggregation degrees for the different PMMA matrices were in the following order: LiCF_3_SO_3_ > LiCOOCF_3_ > LiClO_4_.

**Figure 3:**
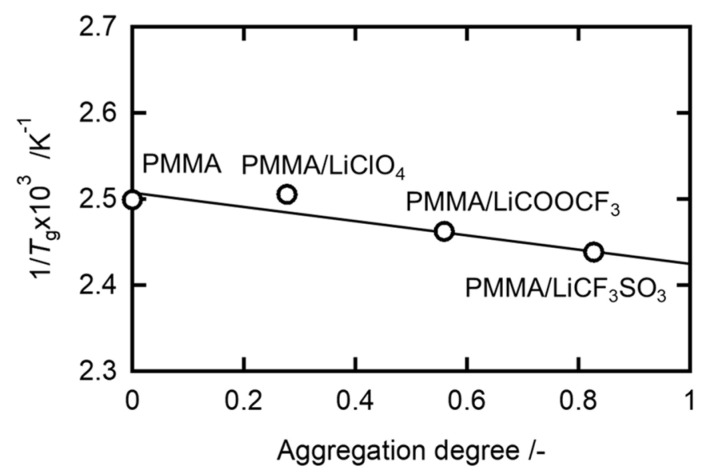
The inverse dependence of *T_g_* on the aggregation degree for the PMMA/salt samples with [Li]/[C=O] = 0.07.

As shown in Figure 3, the reciprocal *T_g_* was proportional to the aggregation degree, according to the following empirical relationship:

(1) $$\frac{1}{T_{g}}=\frac{1-\phi }{T_{g}^{0}}$$

where $T_{g}^{0}$ is the glass transition temperature of pristine PMMA, and *φ* is the aggregation degree of the salt anions, estimated from the fraction of non-free anions. The inverse proportionality of *T_g_* implies the homogeneous dispersion of the added salt in the host PMMA matrix. It is likely that the molecular interaction between the polar groups of PMMA and the anion aggregates suppresses the overall segmental motion of PMMA. The effects of the cohesive force between the anions of the added salt are shown in the Supporting Information. It is interesting to note, however, that a chemical crosslinking process reduces the *T_g_* of PMMA, as demonstrated by Pásztor et al. [25].

### 2.2. Effects of Anion Sizes of the Salts

**Figure 4:**
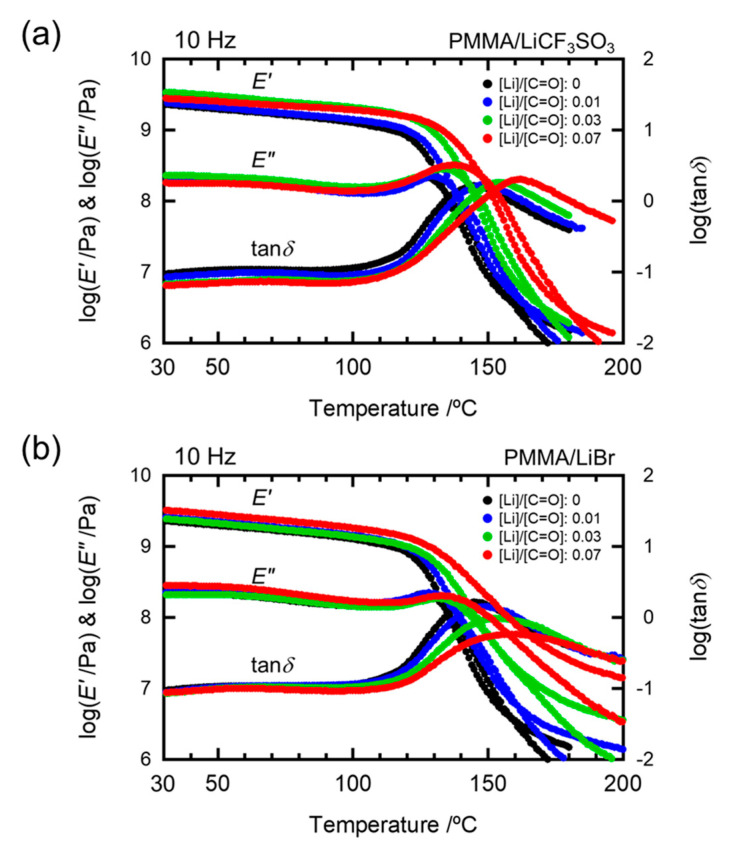
Temperature dependences of dynamic mechanical spectra for (**a**) PMMA/LiCF_3_SO_3_ and (**b**) PMMA/LiBr.

We investigated the effects of the anion sizes of the salts on the formation of the salt aggregates by comparing the doping effects of LiCF_3_SO_3_ and LiBr on the *T_g_* of the PMMA matrix. As shown in Figure 8, there are also no significant peaks derived from the salt in the XRD pattern of PMMA doped with LiBr. Figure 4 shows the temperature dependence of the dynamic mechanical spectra, tan*δ*, for the PMMA/LiCF_3_SO_3_ and PMMA/LiBr samples for [Li]/[C=O] in the 0–7 mol% range. The *T_g_* values of PMMA/LiCF_3_SO_3_ monotonously shifted toward higher temperatures with increasing salt concentrations. This behavior was also observed for the LiCOOCF_3_ and LiClO_4_ doped samples. On the other hand, the doping of LiBr broadened the glass-transition peak as the concentration of LiBr increased [18,22].

**Figure 5:**
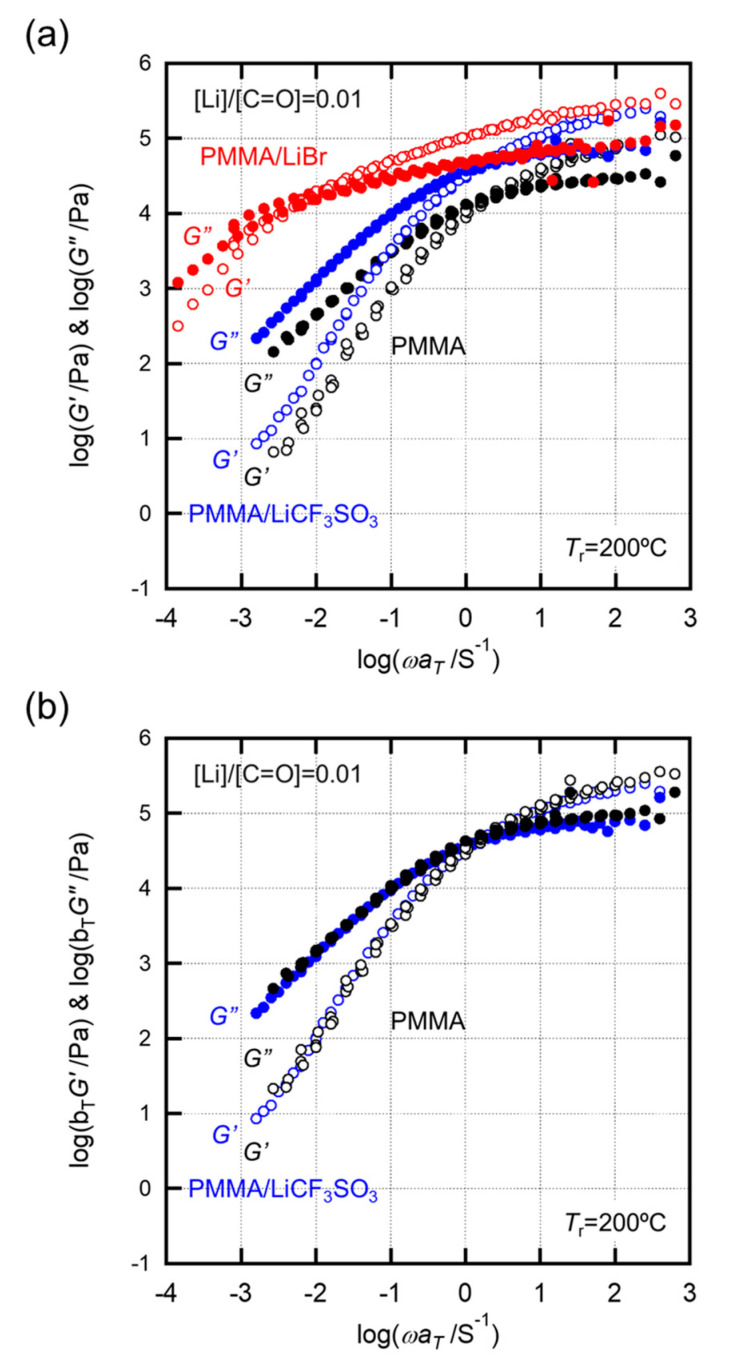
(**a**) Storage and loss moduli of PMMA/LiCF_3_SO_3_ and PMMA/LiBr with [Li]/[C=O] = 0.01 plotted against the frequency reduced to 200 °C. (**b**) The longitudinal shift of PMMA/LiCF_3_SO_3_ toward PMMA.

Figure 5 shows the rheological behavior, from rubbery to flow regions for both LiCF_3_SO_3_^−^ and LiBr-doped samples. The averaged relaxation times, which were estimated from the crossover points of *G’* and *G”* master curves, were 0.40 s for pristine PMMA, 0.63 s for PMMA/LiCF_3_SO_3_, and 180 s for PMMA/LiBr [18,22]. Furthermore, as shown in Figure 5b, the master curve of PMMA can be successfully superimposed on the curve of PMMA/LiCF_3_SO_3_ by shifting to a higher modulus on the longitudinal axis under a slight horizontal shift on the frequency axis. The horizontal shift results from the ratio of average relaxation times. Thus, the addition of the conventional LiCF_3_SO_3_ salt shifted the overall segmental motion of the PMMA chains toward higher frequencies, based on a simple thermo-rheological process. Note that the longitudinal shift in PMMA/LiCF_3_SO_3_ indicates an overall increase in the modulus, which can be considered as a filler effect of the salt aggregates homogeneously dispersed in the PMMA matrix. This is because the cohesive force of the salts is superior to the intermolecular interactions with the PMMA chains.

**Figure 6:**
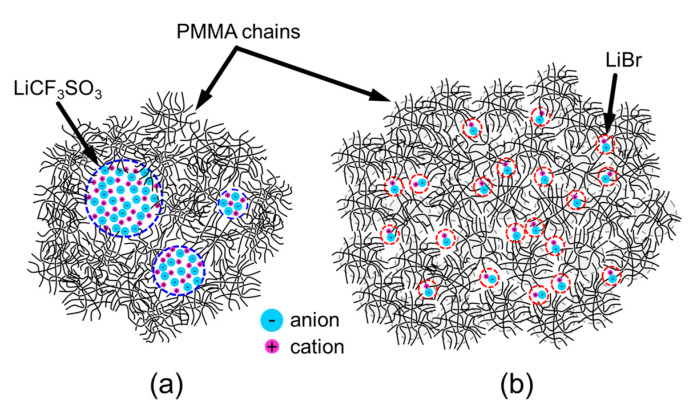
A schematic of the models of (**a**) PMMA/LiCF_3_SO_3_ and (**b**) PMMA/LiBr with conventional lithium salts and lithium halide.

On the other hand, the relaxation time spectra of PMMA/LiBr expanded considerably over time. The terminal zone in the *G*’ and *G*” spectra was prolonged and suppressed the melt-flow behavior of the PMMA chains. Importantly, the *G*’ and *G*” curves of pristine PMMA could not be superimposed on the curves of PMMA/LiBr. This implies that the LiBr salts effectively expanded the melt-flow regions of PMMA. Therefore, the prolonged terminal zone in PMMA/LiBr suggests that the PMMA chains were associated with the Br anions, and the associated PMMA chains exhibited an increase in molecular weight and/or gelation, as seen in ionomers. In addition, it was confirmed using a conventional methanol-based immersion test that the association in PMMA/LiBr is owing to physical (rather than chemical) linkages. On the other hand, and as described above, the LiCF_3_SO_3_ salts aggregate in the PMMA matrix where the salt aggregates suppress the overall segmental mobility of the PMMA chains. The structural images are shown in Figure 6.

**Figure 7:**
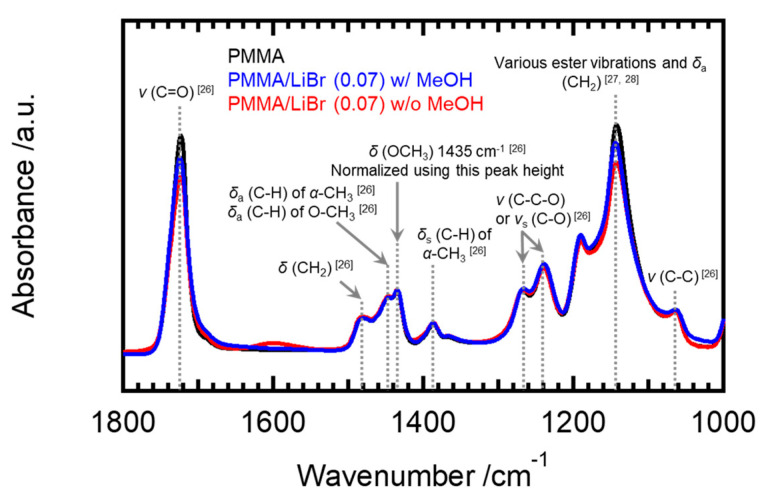
ATR spectra normalized to the peak height at the wavenumber of 1435 cm^−1^, for PMMA (black), PMMA/LiBr after rinsing with methanol (blue), and PMMA/LiBr (red), for wavenumbers in the 1000–1800 cm^−1^ range. Their concentrations of LiBr are [Li]/[C=O] = 0.07.

Figure 7 compares the ATR spectra of pristine PMMA and PMMA/LiBr with 7 mol% salt sheets, with and without rinsing with methanol. 

The positions of the absorption bands in the ATR spectra and their assignments are summarized in Table S2 in the Supporting Information. The ATR spectra were normalized to a peak height at 1435 cm^−1^, assigned to the symmetric stretching vibration mode of OCH_3_ [26]. Figure 7 [27,28] shows that only the intensity of the 1724 cm^−1^ band ascribed to carbonyl groups decreased after adding LiBr but increased owing to the methanol rinsing, indicating that methanol washing weakened the association between carbonyl groups and the salt anions. Interestingly, a broad peak appeared in the 1500–600 cm^−1^ range for PMMA/LiBr, as shown in Figure 7. According to a classical coupled vibration model between C=O and lithium, the vibration mode of the coupling interaction is expected in the 1400–1500 cm^−1^ range. Details of the calculations are provided in the Supporting Information. This result led us to conclude that strong interactions comparable to covalent bonds, such as single and double bonds, are formed between oxygen atoms and lithium cations. This may be ascribed to the ionic association of PMMA.

## 3. Materials and Methods

### 3.1. Materials and Sample Preparation

PMMA pellets (Acrypet VH; *M*_w_ = 120,000, molecular weight distribution index of *M*_w_/*M*_w_ = 2.2) were commercial grades. LiCF_3_SO_3_ (purity ≥ 98.0%; Kanto Chemical Industry Co., Ltd., Tokyo, Japan), LiCOOCF_3_ (purity 97%; Alfa Aesar, Massachusetts, America), LiClO_4_·3H_2_O (purity 97%; Nacalai Tesque, Inc., Kyoto, Japan), and LiBr (purity ≥ 98.0%; Tokyo Chemical Industry Co., Ltd., Tokyo, Japan) were purchased and used without any additional purification. The salt content was estimated as the molar ratio of lithium ions (Li) to PMMA carbonyl groups (C=O). The PMMA/salt films were cast using a mixture of dichloromethane and methanol at a weight ratio of 9:1 for 1 h. The salt concentration was fixed at 0.07 molar ratio of lithium cations with respect to the PMMA ester groups for each sample, except for the measurements of viscoelastic properties. These LiClO_4_, LiCOOCF_3_, and LiCF_3_SO_3_ salts used in this work were relatively soluble with PMMA; on the other hand, LiBr was less soluble. In order to clarify the additive effects of LiCF_3_SO_3_ and LiBr, we used the maximum concentration of LiBr, which was 7 mol%.

After drying in a fume hood, the samples were dried at 160 °C for 30 h to evaporate the residual solvents. Finally, the PMMA/salt sheets with a thickness of approximately 300 µm were obtained by compression molding at 200 °C and 30 MPa for 10 min and were quenched at 25 °C for 3 min. The ionic conductivity values of these sheets were too low to be precisely estimated.

### 3.2. Characterization

X-ray diffraction (XRD) measurements of the prepared samples were performed using a SmartLab diffractometer (Rigaku Co., Ltd., Tokyo, Japan) to estimate the miscibility of the added salts in the samples. The X-ray radiation source was Cu K*α*, and an X-ray tube voltage of 40 kV and a current of 30 mA were used. The diffraction angle ranged from 5° to 80°. The scan step and duration were 0.1° and 1 s, respectively.

**Figure 8:**
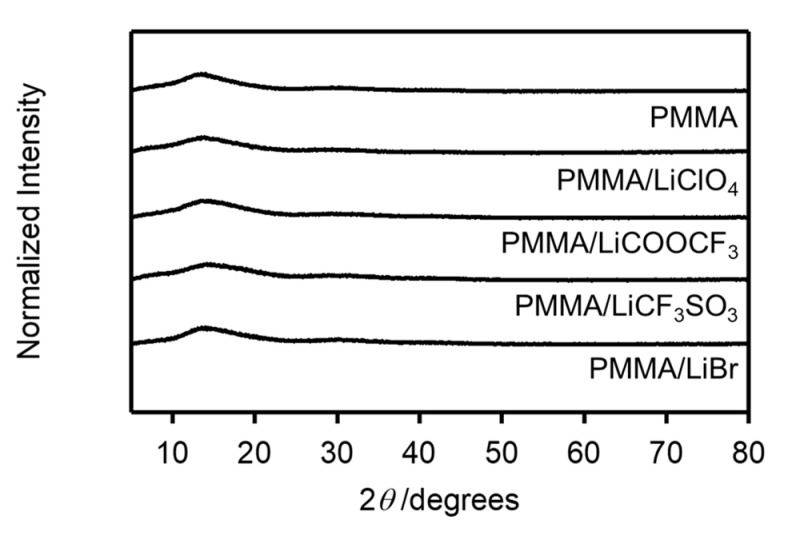
XRD patterns of PMMA/salt samples with [Li]/[C=O] = 0.07.

The XRD patterns are shown in Figure 8. The absence of crystallization in the added salts suggests that the salts were uniformly dispersed in the PMMA matrix.

Ultraviolet-visible (UV-vis) absorption spectra were obtained using V650 (JASCO Co., Ltd., Tokyo, Japan) for wavelengths in the 400–700 nm range. The UV-vis spectra of the prepared samples showed that the transmittance values of the samples with salt concentrations of 7 mol% were above 80%, except for PMMA/LiBr (Figure S1).

### 3.3. Measurements

Dynamic mechanical analysis was performed using Rheogel-E4000 (UBM Co., Ltd., Kyoto, Japan). Rectangular specimens with the 5 mm width and 20 mm length were cut out from the sheets with a thickness of approximately 300 μm. The temperature dependences of the tensile storage modulus (*E*’) and loss modulus (*E*”) were measured for temperatures in the 30–200 °C range with an initial chuck distance of 10 mm. The frequency and the heating rate were 10 Hz and 2 °C/min, respectively. The frequency dependences of the oscillatory shear moduli *G*’ and *G*” were measured using a cone-and-plate rheometer in the temperature range of 200–240 °C using Rheogel-E4000 (UBM Co., Ltd., Kyoto, Japan). Attenuated total reflection (ATR) measurements were performed using Spectrum 100 (PerkinElmer Co., Ltd., Kanagawa, Japan) with a KRS-5 ATR prism for evaluating the infrared spectra under a nitrogen flow. The accumulation count was 16, and the resolution was 4 cm^−1^.

## 4. Conclusions

LiCF_3_SO_3_, LiCOOCF_3_, and LiClO_4_, which are conventionally used as lithium electrolytes, and an alkali halide, LiBr, were incorporated into PMMA. In the case of LiCF_3_SO_3_, LiCOOCF_3_, and LiClO_4_, the aggregates were homogeneously dispersed in the PMMA matrix, and these salts thermo-rheologically shifted the segmental mobility of PMMA toward lower temperatures. In addition, the Li salts effectively acted as fillers in the PMMA matrix, suggesting that they can be used as stiffening agents. On the other hand, the alkali halide molecules acted as physical cross-linkages, leading to the ionic association of the PMMA chains and resulting in the expansion of the terminal relaxation time spectra of PMMA into the flow region. The addition of these lithium salts enables controlling the melt flow behavior of PMMA.

## Figures and Tables

**Table 1:** The values of *T_g_* for the prepared PMMA/salt samples with [Li]/[C=O] = 0.07.

Sample Code	*T_g_*/°C
PMMA	127
PMMA/LiClO_4_	126
PMMA/LiCOOCF_3_	133
PMMA/LiCF_3_SO_3_	137
PMMA/LiBr	133

**Table 2:** The contribution ratios of the structures of anionic species (ClO_4_^−^, CF_3_SO_3_^−^, and COOCF_3_^−^) calculated using the area ratios of the ATR spectra peaks [22]. Their concentrations of lithium salts are [Li]/[C=O] = 0.07.

Sample Code	Peak Area/%		
	PMMA/LiClO_4_	PMMA/LiCOOCF_3_	PMMA/LiCF_3_SO_3_
Free	72.3	44.1	17.3
Ion pair	27.7	40.8	42.7
Triple ion	―	5.7	40.0
Aggregates	―	9.3	―

## Data Availability

The data presented in this study are available in supplementary material.

## Supplementary Materials

The following are available online. Figure S1: UV-Vis spectra of PMMA/salt samples with [Li]/[C=O] = 0.07 and PMMA/LiBr with [Li]/[C=O] = 0.01. PMMA/LiBr with 1 mol% of the salt concentration showed low transmittance below 30%. Figure S2: ATR spectra of PMMA/salt samples in the range of the stretching vibration mode of carbonyl groups with [Li]/[C=O] = 0.07. Figure S3: Relationship between the *T**_g_* of the PMMA/salt samples and cohesive energies of salts, for [Li]/[C=O] = 0.07. Figure S4: Three masses are connected by a Hookean spring to each other. This system describes coupled motion along one dimension. Table S1: Functional groups and molar attraction constants of the salts. Table S2: Wavenumbers and assignments of infrared absorption bands of PMMA.

## Sample Availability

Samples of the compounds are not available from the authors.
