# Supplementary material for: Additive Effects of Lithium Salts with Various Anionic Species in Poly (Methyl Methacrylate)
Source: Molecules. 2021 Jul 5;26(13):4096. doi: 10.3390/molecules26134096 (PMC8271803; doi:10.3390/molecules26134096)
Supplement: Supplementary file 1 [file molecules-26-04096-s001.zip › molecules-1278365-supplementary.pdf]

molecules

Supporting Information

Additive effects of lithium salts with various anionic species in poly(methyl methacrylate)

Asae Ito and Koh-hei Nitta*

Polymer physics laboratory, Institute of Science and Engineering, Kanazawa University, Kakuma Campus, Kanazawa, Ishikawa, 920-1192, JAPAN

1. Characterization of the PMMA/salt samples

Figure S1 shows the UV-vis spectra of the PMMA/salt samples, where the transmittance values of the samples with 7 mol% of the salt concentrations were above 80%., except for PMMA/LiBr (Figure S1). PMMA/LiBr (1 mol%) yielded a yellow-colored film, and the transmittance dropped below 50 %. Figure S2 shows the ATR spectra in the range of the stretching vibration mode of carbonyl groups, for wavenumbers in the 1650–1780 cm^{-1} range.

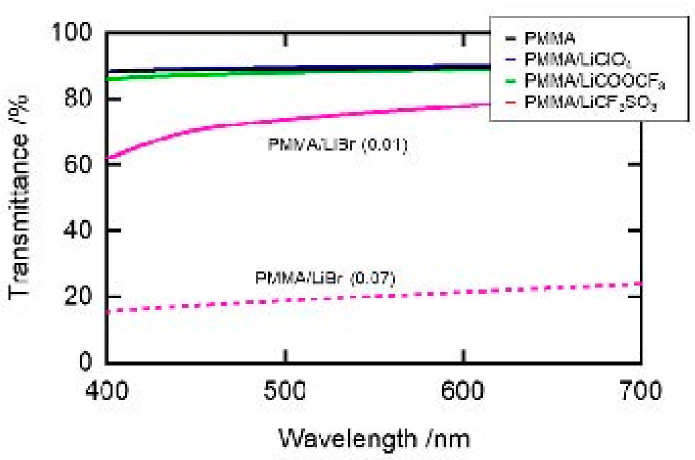

Figure S1 UV-Vis spectra of PMMA/salt samples with [Li]/[C=O] = 0.07 and PMMA/LiBr with [Li]/[C=O] = 0.01. PMMA/LiBr with 1 mol% of the salt concentration showed low transmittance below 30%.

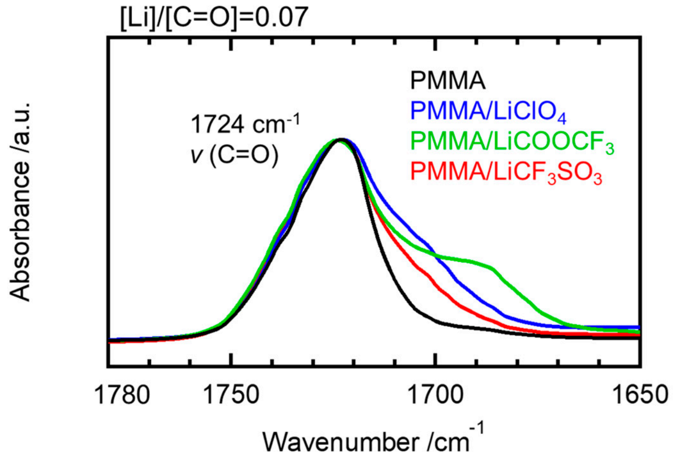

Figure S2 ATR spectra of PMMA/salt samples in the range of the stretching vibration mode of carbonyl groups with [Li]/[C=O] = 0.07.

2. Solubility parameter of salt species

We plotted the values of T_g against the cohesive energy of salts estimated using the atomic group contribution method, to validate the results in Figure 4. The cohesive force between the salts increased with T_g of the PMMA matrix, as shown in Figure S3.

Thus, we calculated the solubility parameters by estimating the cohesive energy of the salts using the atomic cluster contribution method and plotted the results against T_g. The molar gravitational constants for each functional group[1] of each salt used in this calculation are listed in Table S1.

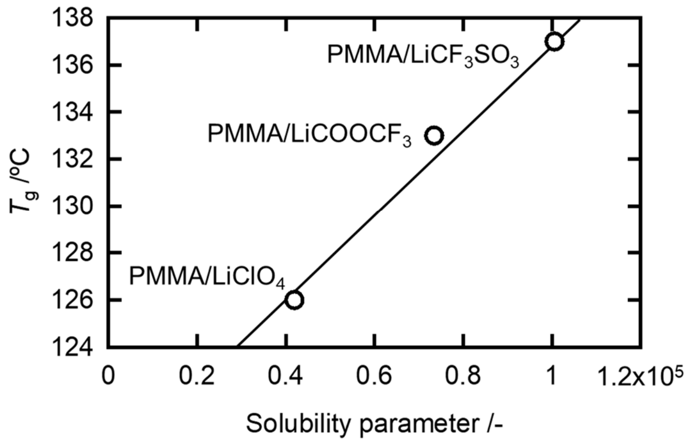

Figure S3 Relationship between the T_g of the PMMA/salt samples and cohesive energies of salts, for [Li]/[C=O] = 0.07.

Table S1 Functional groups and molar attraction constants of the salts.

Functional group	Molar Attraction Constant ($MPa^{1/2}$ · cm^3/mol)
-COO	569
$-CF_3$	496
SO_3	721
-Cl	526
-O-	100

3. A coupled vibration mode model for IR spectra

We considered a bead-spring model assuming a coupling interaction between the vibration of carbonyl groups and Li cations (Figure S4). We restricted the motion to the line connecting the masses, so the system corresponds to the stretching vibration modes of IR spectra and has only three degrees of freedom, represented by the coordinates x_1, x_2, and x_3. Each coordinate was measured from the equilibrium position. As a result, the determinant of the following matrix provided a nontrivial solution for the system of simultaneous motion equations:

$$\begin{vmatrix} \lambda - \omega_{11}^2 & \omega_{11}^2 & 0 \\ \omega_{12}^2 & \lambda - (\omega_{11}^2 + \omega_{22}^2) & \omega_{22}^2 \\ 0 & \omega_{23}^2 & \lambda - \omega_{23}^2 \end{vmatrix} = 0 \quad \text{(S1)}$$

Here, the angular frequency is $\omega_{ij} = \sqrt{N_A k_i / M_j}$, where N_A is the Avogadro number and M_j is the molecular weight of each particle.

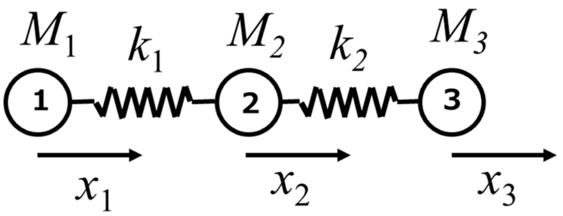

Figure S4 Three masses are connected by a Hookean spring to each other. This system describes coupled motion along one dimension.

Here, particles 1, 2, and 3 are C, O, and Li atoms, respectively. The spring constant k_1 was estimated from 1724 cm^{-1}, which is the IR band of the carbonyl group, giving $k_1 = 12 \times 10^2$ N/m. Assuming that the spring constant k_2 is in the 1×10^2 –10×10^2 N/m range, corresponding to the single to double bonds, a novel vibration mode appeared in the 1400–1500 cm^{-1} range.

In addition, we present the assignments of infrared absorption bands of PMMA[2–4].

Table S2 Wavenumbers and assignments of infrared absorption bands of PMMA[2–4].

Wavenumbers (cm^{-1}) in this result	Wavenumbers (cm^{-1}) reported in reference [2-4]	Assignments [2-4]
2994	2995 [2]	ν_a (C-H) of α-CH_3, ν_a (C-H) of OCH_3 [2]
2950	2948 [2]	ν_s (C-H) of OCH_3 with ν_s (C-H) of α-CH_3, and ν_a (CH_2) [2]
2924	2915-2920 [2]	Combination band involving OCH_3 and ν_s (CH_2) [2]
1724	1730 [2]	ν (C=O) [2]
1485	1483 [2]	δ (CH_2) [2]
1448	1452, 1465 [2]	δ_a (C-H) of α-CH_3, δ_a (C-H) of O-CH_3 [2]
1435	1438 [2]	δ (C-H) of OCH_3 [2]
1387	1388 [2]	δ_s (C-H) of α-CH_3 [2]
1268	1270 [2]	ν (C-C-O) or ν_s (C-O) [2]
1239	1240 [2]	
1190	1190 [2, 3], 1192 [4]	Under discussion [2-4]; possibly ν (C-O-C) [3]
1143	1150 [3], 1148 [4]	Combination of delocalized modes of various ester vibrations and CH_2 rocking modes [3, 4]
1064	1063 [2]	ν (C-C) [2]

References

1. Ogawa, T. Numerical Prediction of Solubility Parameter using Molar Attraction Constant. *J. Adhes. Soc. Japan* **2017**, *53*, 129–136, doi:10.11618/adhesion.53.129.
2. Willis, H.A.; Zichy, V.J.I.; Hendra, P.J. The laser-Raman and infra-red spectra of poly(methyl methacrylate). *Polymer (Guildf).* **1969**, *10*, 737–746, doi:10.1016/0032-3861(69)90101-3.
3. Brinkhuis, R.H.G.; Schouten, A.J. Thin-film behavior of poly(methyl methacrylates). 2. An FT-IR study of Langmuir-Blodgett films of isotactic PMMA. *Macromolecules* **1991**, *24*, 1496–1504, doi:10.1021/ma00007a010.
4. Havriliak, S.; Roman, N. The infra-red absorption characteristics of syndiotactic poly(methyl methacrylate) from 1050 cm-1 to 1300 cm-1. *Polymer (Guildf).* **1966**, *7*, 387–400, doi:10.1016/0032-3861(66)90054-1.
